# Probing Cellular and Molecular Mechanisms of Cigarette Smoke-Induced Immune Response in the Progression of Chronic Obstructive Pulmonary Disease Using Multiscale Network Modeling

**DOI:** 10.1371/journal.pone.0163192

**Published:** 2016-09-26

**Authors:** Zhichao Pan, Haishan Yu, Jie-Lou Liao

**Affiliations:** Department of Chemical Physics, University of Science and Technology of China, 96 Jinzhai Road, Hefei, Anhui Province, 230026, People’s Republic of China; University of Rochester Medical Center, UNITED STATES

## Abstract

Chronic obstructive pulmonary disease (COPD) is a chronic inflammatory disorder characterized by progressive destruction of lung tissues and airway obstruction. COPD is currently the third leading cause of death worldwide and there is no curative treatment available so far. Cigarette smoke (CS) is the major risk factor for COPD. Yet, only a relatively small percentage of smokers develop the disease, showing that disease susceptibility varies significantly among smokers. As smoking cessation can prevent the disease in some smokers, quitting smoking cannot halt the progression of COPD in others. Despite extensive research efforts, cellular and molecular mechanisms of COPD remain elusive. In particular, the disease susceptibility and smoking cessation effects are poorly understood. To address these issues in this work, we develop a multiscale network model that consists of nodes, which represent molecular mediators, immune cells and lung tissues, and edges describing the interactions between the nodes. Our model study identifies several positive feedback loops and network elements playing a determinant role in the CS-induced immune response and COPD progression. The results are in agreement with clinic and laboratory measurements, offering novel insight into the cellular and molecular mechanisms of COPD. The study in this work also provides a rationale for targeted therapy and personalized medicine for the disease in future.

## Introduction

Chronic obstructive pulmonary disease (COPD) is characterized by airflow limitation caused by destruction of the lung parenchyma and/or airway obstruction [1–3]. COPD is currently the third leading cause of death worldwide and poses a major public health burden globally [4]. Moreover, COPD is associated with the development of lung cancer [5]. There is no cure available for COPD and current drugs are mainly effective in improving symptoms and exacerbations but generally do not slow down the progression of the disease [6]. Therefore, it is important to understand the cellular and molecular mechanisms of COPD for developing effective treatments of the disease.

COPD is a chronic inflammatory disease caused by inhalation of toxic particles and gases, mostly cigarette smoke (CS) [1–3,7]. Despite the fact that CS is the major risk factor for COPD, many chronic smokers maintain normal lung function (so-called resistant smokers) [2], so do some smokers even after more than 40 pack years of smoking [8], while only ~20–30% of chronic smokers develop the disease [1, 2,7,9]. This suggests that the susceptibility of smokers to COPD can vary significantly [1, 2, 8, 9]. However, the cellular and molecular basis for the disease susceptibility remains to be elucidated albeit genetic or environmental factors may play a role [1, 2]. As chronic cigarette smokers with normal lung function also have increased pulmonary inflammation, this inflammation seems to be magnified in COPD. Understanding of the amplification of inflammation is not yet complete [1]. Cigarette smoking cessation is considered currently as the most important intervention to reduce COPD progression [10]. While quitting smoking can prevent the COPD progression in some patients, who are referred as (reversibly) susceptible smokers, cigarette smoking cessation fails to slow or preclude the COPD progression in others (referred as severely susceptible smokers) [2, 11]. The precise understanding of different effects of smoking cessation has not yet been fully achieved [1–2].

The CS-induced inflammatory response in COPD progression involving both innate and adaptive immunity [1, 2] is mediated via a complex network that encompasses multiple immune cell types, molecular mediators and lung tissues. Several different types of immune cells and molecular mediators are found to accumulate in the lungs of patients with COPD [1–3, 5–7, 12]. Important immune cells include macrophages, neutrophils, dentritic cells, and T lymphocytes and molecular mediators include cytokines, chemokines, and protein proteases such as metalloproteases (MMPs). There exists an enormous amount of literature regarding these individual network elements. However, little is known about combined interactions between these elements or the associated pathways in the network. In particular, while COPD progression is a multistage and dynamic process, studies on the temporal sequence of inflammation in the disease are lacking [2]. It is not clear how immune cells and molecular mediators are dynamically linked and which of these elements are determinants in the disease progression. This is particularly important for identification of biomarkers in the disease [6, 13–17]. For example, the levels of proinflammatory cytokines, TNF-α and IL-1β are increased in the lungs of COPD patients and were suggested as potential targets [6]. However, inhibition of TNF-α or IL-1β has been proved to be unsuccessful in clinical trials of patients with COPD [6]. A pilot study on patients with COPD revealed no change in levels of inflammatory markers following inhibition of TNF-α [18], but the underlying reason remains to be elucidated [6, 19]. Recent studies have shown that the levels of IL-6 as well as the associated proteins, C-reactive protein (CRP) and fibrinogen, are significantly increased in COPD patients compared to those in smokers with normal lung function and healthy non-smokers [16]. IL-6 is considered to be a potential biomarker for COPD, but the detailed mechanism of IL-6 action in the disease progression is not yet fully understood.

In this study, we develop a network model for CS-induced immune response in the lung to address the issues described above. Our model analysis identifies several positive feedback loops that play a determinant role in the CS-induced immune response and COPD progression, providing novel insight into the cellular and molecular mechanisms of the disease.

## Materials and Methods

The associated immune system is highly complicated as it involves many mediator molecules, multiple immune cell types, and lung tissues, presenting a challenge for quantifying the dynamics of CS-induced COPD. For simplicity in the following discussion, an immune response network model is constructed by treating important immune cells, cytokines, and lung tissues as network nodes. There are two types of inputs initiated from a node: a positive or an up-regulation input (denoted by→) indicates that an increasing of the concentration of the tail node will lead to an increasing of that of the head node or an up-regulation of the process when the input arrow ends at an edge between two nodes, and vice versa for a negative or a down-regulation (inhibition) input (denoted by ┫).

A network model for CS-induced immune response in COPD progression in this work is developed based on established literature knowledge. Upon exposure to CS, immature alveolar macrophages (M0) are classically activated directly by the CS particulate phase [12] and polarized to the inflammatory phenotype (M1) in the lung [20, 21]. The M1 cells produce inflammatory cytokines such as TNF-α, which activates M1 conversely, IL-6 and IL-12 [22, 23]. M1 can cause tissue damage (TD) in the lung by releasing reactive oxygen species (ROS) leading to oxidative stress, proteases such as macrophage elastase and metalloproteases (MMPs) to ingest pathogens and apoptotic cells, and chemokines to recruit neutrophils into the lung [24–26]. Neutrophils, which are short-lived and subsequently cleared by macrophages [20], can also cause TD in a manner similar to macrophage [25]. Furthermore, the tissues damaged by M1 can produce elastin fragments (EFs) as strong attractors to recruit monocytes (precursors of macrophages, M0) into the lung from circulation [27].Subsequently, these M0 cells are differentiated into M1, thus forming a positive feedback loop, M1→TD→M1. In addition, TD can also be caused directly by CS [7].

TD is an early source of IL-4 production (by basophils and mast cells in TD) that leads to alternatively activated macrophages (M2) [26, 28]. M2 can release IL-10, which can conversely activate M2, and transforming growth factor, TGF-β [2]. While IL-10 is a potent anti-inflammatory cytokine that down-regulates almost all important proinflammatory and TD-related processes, TGF-β is a multi-functional growth factor. In small airways, TGF-β is a potent inducer for extracellular matrix target genes such as collagens, and fibroblast proliferation and activation which both are key events in the fibrogenic process [29]. However, in the lung parenchyma, TGF-β down-regulates tissue damage through inhibition of MMP-12 and MMP-9 [29].

Dendritic cells (DCs) are antigen-presenting and play a critical role in linking the innate to the adaptive immune response [1, 30]. Immature dendritic cells (DC0) near the epithelial surface are activated directly by CS or dangerous signals generated from TD [7]. Dendritic cells undergo a maturation process and migrate towards the local lymph nodes. Naïve, quiescent T cells cannot enter the lung parenchyma. But once activated by matured DC, they can move into the lung and differentiate into Th1, Th2, Th17, T-regulatory (Treg) and CD8^+^T cells, which are proliferated by themselves, in their corresponding cytokine environments [31, 32], e.g., in the presence of IL-12 secreted by M1 (as well as DC), naïve CD4^+^T cells (Th0) differentiate into T helper 1 (Th1) cells [33–35]. The Th1 cells secrete IFN-γ to up-regulate the polarization process from M0 to M1 [22, 36]. A multi-node positive feedback loop, M1→IL12→Th1→IFN-γ→M1, is thus formed. In contrast to Th1, Th2 is polarized from Th0 in the presence of IL-4. Th2 produces IL-13 and IL-4, release of which further enhances the production of IL-10 and TGF-β by M2. In the presence of TGF-β, Th0 cells differentiate into Treg, which secrete IL-10 [22, 36]. TGF-β and IL-6 (rather than IL-23) together induce Th17 differentiation, leading to the production of IL-17 [37–41]. While IL-17 acts on epithelial cells to recruit neutrophils to cause tissue damage further, the activated epithelial cells in TD secrete IL-6, forming a positive feedback loop, IL-6→Th17→IL-17→TD→IL-6 [39]. Th17 cells also produce IL-21 for the differentiation of CD8^+^T cells from naïve CD8 cytotoxic T lymphocytes (T0) [37, 42]. While CD8^+^T cells produce IFN-γ to enhance the M1 inflammatory activities, they also release granzyme B and perforins, causing apoptosis/necrosis of targeted cells and leading to TD further [37]. In addition, IL-6 can down-regulate the activation of Treg that secretes IL-10 to inhibit Th17 [43]. Consequently, a positive feedback loop, IL-6┫Treg→IL-10 ┫Th17→IL-17→TD → IL-6, is formed.

**Fig 1:**
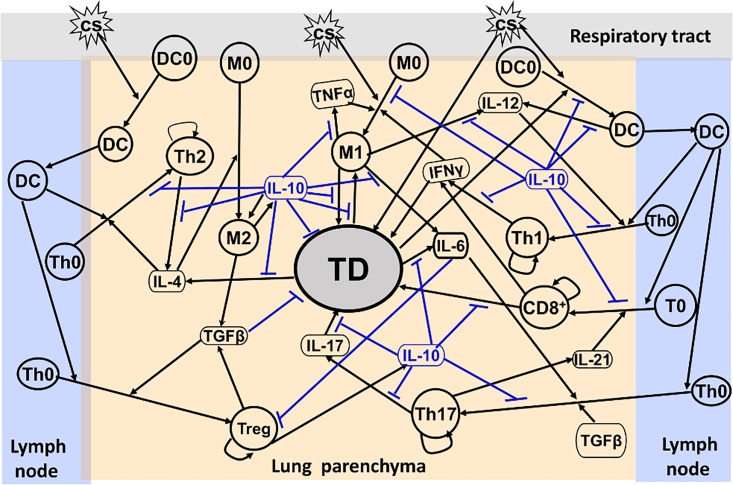
Network model for CS-induced immune response. Interactions between various nodes that represent cytokines, immune cells, and TD are described (for detailed description, see text).

As proposed earlier, the immune cells, cytokines and TD discussed above can be treated as network nodes. The aforementioned interactions between these nodes are then integrated into the network model shown in Fig 1 in the next section. The constructed network bears a multiple timescale feature. Cytokine regulation of cell function through signal transduction usually occurs on a sub-second timescale, for example, whereas cell production of cytokines takes minutes to hours [44]. The cytokine regulation activity can thus be considered to be at steady state in the equations that describes the slow timescale activities of the cells. In this way a positive or a negative input can be modeled using an increasing or decreasing Hill function, respectively [44,45]. The dynamics of the cytokines, the immune cells and TD can thus be described using a set of ordinary differential equations (ODEs). In this study, the system of ODEs is solved numerically using MATLAB (version R2013a Mathworks) with a variable order and multistep solver, ode15s. MATLAB is also used to plot the simulation data to generate the figures presented below.

## Results

### Network Model

In the present network model shown in Fig 1, M1, DC, Th1, CD8^+^T and Th17 cells along with their corresponding cytokines, TNF-α, IL-6, IL-12, IFN-γ, and IL-17 form multiple proinflammatory pathways, whereas M2, Th2 and Treg cells with the associated cytokines, IL-4, TGF-β, and IL-10, form anti-inflammatory/regulatory pathways. The inflammatory and anti-inflammatory/regulatory pathways are interlinked with each other through several nodes representing molecular mediators such as IL-6, TGF-β and IL-10 (Fig 1). These pathways eventually converge at the TD node that represents the tissue damage. Here, we focus on the immunologic aspects of COPD and the TD node is highly coarse-grained, involving neutrophil-induced tissue damage, epithelial and endothelial cell injury and extracellular matrix degradation etc. As discussed above, for example, M1 produces chemokines to recruit neutrophils (not shown in Fig 1) for the release of neutrophil elastase and ROS, contributing to TD. The representation of this process is included indirectly in the M1→TD motif shown in Fig 1. In addition, while M1 secretes MMPs to cause TD, TD also produces EFs to recruit monocytes for generating M1. This process is represented in the M1→TD→M1 positive feedback loop where MMPs and EFs are included indirectly (Fig 1). As TD is the major feature of CODP in the lung parenchyma, the dynamics of TD is used to characterize the progression of COPD in this work.

As mentioned above, the dynamics of the network elements can be described by a set of ODEs. Here, the ODEs involve the following 18 variables: M_1_, M_2_, D_C_, T_1_, T_2_, T_8_, T_17_, and T_g_ represent the densities of M1, M2, DC, Th1, Th2, CD8^+^T, Th17, and Treg cells (in units of cell numbers in a cubic millimeter of tissue), respectively, whereas I_4_, I_6_, I_10_, I_12_, I_17_, I_21,_ I_α_, I_γ_, and I_β_ denote concentrations of the cytokines, IL-4, IL-6, IL-10, IL-12, IL-17, IL-21,TNF-α, IFN-γ, and TGF-β. The variable, T_D_, is defined as a ratio (in terms of a percentage) of damaged tissue to the whole lung parenchymal tissue (normal tissue plus damaged tissue). This T_D_ definition is similar to that of destructive index (DI) [46–47] and can be measured experimentally. While a T_D_ value of 0–30% is considered normal, a T_D_ value larger than 30% is associated with COPD [48].

### Immune Cell Dynamics

The M1 population originates from a constant source of M0 [49] whose differentiation is stimulated by the external stimulus, CS [7]. The M0 differentiation process, which is inhibited by I_10_, is up-regulated by I_γ_ and I_α_ at maximal rate k_2_ [50]. As shown in Fig 1, TD also increases the M1 population and this process is also down-regulated by I_10_. Equation for describing the population dynamics of M1 with a decay rate, d_M1_, is then given by

dM1dt=k1S11+(I10K1)2+k2(IγK2)2+(IαK3)21+(IγK2)2+(IαK3)2+(I10K4)2+k3TD11+(I10K5)2−dM1M1, (1)

where $k_{1}=k_{1}^{'}M_{0}$, $k_{2}=k_{2}^{'}M_{0}$ ($k_{1}^{'}$ and $k_{2}^{'}$ are rate constants), and the regulation of the M_0_→M_1_ differentiation process is characterized by the Hill function whose coefficients are often chosen to be 2 to allow sufficient nonlinearity [45]. In Eq 1, S is cigarette smoking intensity, whose values are given below.

M_2_ is differentiated from M_0_ and this process is up-regulated by I_4_ or I_10_ [26]. With a decay rate, d_M2_, the M_2_ dynamics can be described by

dM2dt=k4(I4K6)2+(I10K7)21+(I4K6)2+(I10K7)2−dM2M2 (2)

The population of dendritic cells, D_C_, originates from a constant source of immature D_0_ activated and up-regulated by the external stimulus, S and the damage signal from T_D_ [7]. This process is inhibited by I_10_. With a decay rate coefficient, $d_{D_{C}}$, equation for the D_C_ dynamics is given by

dDCdt=k5S11+(I10K8)2+k6TD11+(I10K9)2−dDCDC, (3)

where $k_{5}=k_{5}^{'}D_{0}$, $k_{6}=k_{6}^{'}D_{0}$.

T cells derive from a constant source of immature T-cells (Th0 or T_0_), which are recruited and activated by D_C_ in combination with specific cytokines as mentioned previously. For T_1_, T_2_, T_8_, and T_17_, the associated T-cell differentiation processes are down-regulated by I_10_. With inclusion of a decay rate, equations describing the dynamics of these T-cell populations can be expressed by

dTidt=ki(IiKTi)21+(IiKTi)2+(I10KTi,10)2+kipTi11+(I10KTi,I10p)2−dTiTi (4)

where T_i_ represents T_1_, T_2_, or T_8_, k_i_ = k_7_, k_8_, or k_9_; I_i_ = I_12_, I_4_, or I_21_, and k^p^_i_ = k^p^_1_, k^p^_2_, or k^p^_8_, which are the maximum rates for the self-proliferation of T_1_, T_2_, and T_8_, respectively. The T_17_ dynamics, which is up-regulated by both I_6_ and I_β_ and is down- regulated by I_10_, can be described by

dT17dt=k10(IβI6KT17,I6)21+(IβI6KT17,I6)2+(I10KT17,I10)2+k17pT1711+(I10KT17,I10p)2−dT17T17 (5)

Equation describing the T_g_ dynamics, which is up-regulated by I_β_ and inhibited by I_6_, can be written as

dTgdt=k11(IβKTg)21+(IβKTg)2+(I6KTg,I6)2+kgp11+(I10KpTg,I10)2−dTgTg (6)

### Cytokine Dynamics

As shown in Fig 1, the cytokines are secreted from their associated immune cells. In particular, T_D_ also contributes to the I_4_ and I_6_ productions, respectively, as discussed previously. The cytokine secretion processes are often down-regulated by I_10_. Accordingly, the population dynamics of the cytokines can be described by

$$\frac{dI_{i}}{dt}=\sum_{j=1}^{N}k_{i,j}C_{j}f_{j}\left(I_{10}\right)+k_{I_{i},T_{D}}T_{D}f_{j}^{D}(I_{10})-d_{i}I_{i}$$ (7)

where C_j_ is the density of the j-th cell, which produces I_i_ with a maxima rate k_i,j_, $k_{I_{i},T_{D}}$ is the rate constant of the I_i_ production by T_D_, f_j_(I_10_) is the regulation function, i.e., the Hill function

fj(I10)=11+(I10KCj,I10)2 (8)

The Hill function, f^D^_j_ (I_10_), in Eq 7 describes the I_10_ down-regulation of the process for I_4_ or I_6_ production by T_D_ (Fig 1). Thus, for I_4_ and I_6_,

fjD(I10)=11+(I10KIi,I10)2 (9)

whereas f_D_ (I_10_) = 0 for other cytokines, whose population dynamics are governed by Equations A-G given in S1 File. For example, Eqs 10–11 describing the dynamics of I_6_ and I_10_ are given by

dI6dt=kI6,M1M111+(I10KM1,I10)2+kI6,TDTD11+(I10KI6,I10)2−dI6I6 (10)

$$\frac{dI_{10}}{dt}=(k_{I_{10},M_{2}}M_{2}+k_{I_{10},T_{g}}T_{g})\frac{1}{1+{\left(I_{10}/K_{I_{10},I_{10}}\right)}^{2}}-d_{I_{10}}I_{10}$$ (11)

In Eq 10, I_6_ comes from M_1_ [26] and T_D_ [37], respectively. These production processes are down-regulated by IL-10 [50–52]. I_10_ in Eq 11 is produced by M_2_ [26] and T_g_ [36], and can be inhibited by itself [49, 51].

### TD Dynamics

As discussed above, T_D_ can be generated by S [7], M_1_ [24–26] and T_8_ [37], respectively. I_γ_ and I_17_ also cause TD [37]. These TD generation processes are all down-regulated by I_10_ [52]. As mentioned earlier, in the lung parenchyma the process for MMP-induced TD generation is also inhibited by I_β_ [29]. Equation describing the T_D_ dynamics thus is

dTDdt=k12S(1−TD)11+(I10K10)2+k13M1(1−TD)11+(I10K11)2+k14T8(1−TD)11+(I10K12)2+k15(IγK14)2+(I17K15)21+(IγK13)2+(I17K14)2+(I10K15)2+(IβK16)2−dTDTD (12)

where T_D_ is defined by damaged tissue/(normal tissue+damaged tissue)×100% and tissue repair is involved indirectly in the last term in Eq 12.

### Sensitivity Analysis

The parameter values in the above equations were taken or estimated from literature in the following discussion. For those whose experimental data are not available, we performed a global sensitivity analysis to obtain order-of-magnitude estimates. Sensitivity analysis is useful to determine parameters that play a critical role in affecting the model outcome [49]. In this study, a global sensitivity analysis outlined by Marino et al. [53] is applied to assess the sensitivity of the model outcome, T_D_, at t = 4000 day in the stable stage of COPD to variations of all parameters in the model. Baseline values of these parameters are listed in Table A in S1 File. A range of 10% to 200% of the baseline values was specified for each parameter in a way similar to that in ref. 49. All parameters are assumed to be uniformly distributed in their corresponding interval and 2000 samples are generated for each parameter using the Latin Hypercube Sampling method [53]. The partial rank correlation coefficient (PRCC) as well as p-value for each parameter is then calculated. The calculated PPRCs with the p-values smaller than 0.01 are presented in Figure A in S1 File. The values of PRCC range between -1 and +1 with the sign determining whether an increase in the parameter will increase (+) or decrease (-) the T_D_ output.

Our sensitivity analysis shows that a set of parameters including k_13_, k_14_, k_15_, and d_TD_ have relatively large PRCCs (>0.1, Figure A in S1 File), suggesting that TD outcomes are sensitive to these parameters. This set of parameters, whose values can be changed from individual to individual, are associated with different network elements, positive feedback loops and pathways, which are critical in the CS-induced immune response and COPD progression. Therefore, investigation of model outcome changes with variations in these parameters can be of great use to disclose the cellular and molecular mechanisms of COPD as discussed below.

### Simulations of Dynamics of Immune Responses to CS at Different Dose Levels

**Fig 2:**
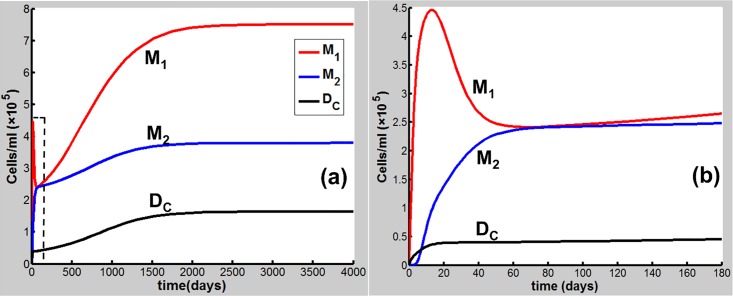
CS-induced (S = 1.67) population dynamics. M_1_, M_2_ and D_C_ of dynamics over a time period of (a) 4000 days and (b) 180 days [the dashed square region in (a)].

**Fig 3:**
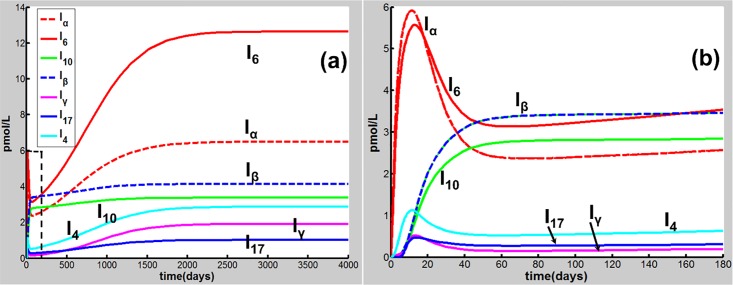
CS-induced (S = 1.67) population dynamics. Dynamics of I_α_, I_6_, I_10_, I_β_, I_γ_, I_17_ and I_4_ over a time period of (a) 4000 days and (b) 180 days [the dashed square region in (a)].

**Fig 4:**
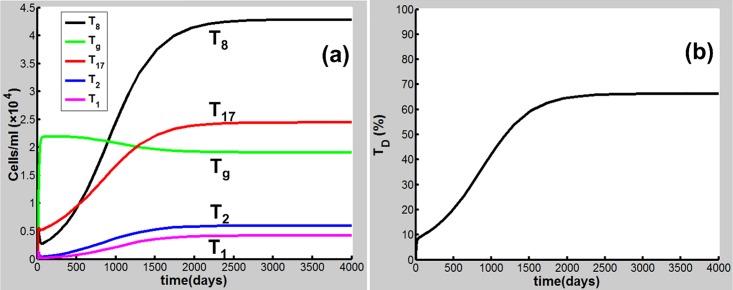
CS-induced (S = 1.67) population dynamics. Dynamics of (a)T_8_, T_g_, T_17_, T_2_, and T_1_, and (b) T_D_.

Eqs 1–12 are applied in the following discussion with the parameters given in Table A in S1 File. The time course of the simulations is marked in days and units of cell populations and cytokine concentrations are in terms of cells per milliliter (cells/ml) and pmol per liter (pmol/L). The initial conditions of the variables for all activated cells and secreted cytokines are set to zero. CS-induced immune response and COPD progression are dependent upon the dose of CS inhalation [54]. Figs 2–4 respectively show the time courses of the changes in the immune cells, cytokines and tissue damage (T_D_) in response to CS at a relatively high level of the cigarette smoking intensity, S (S = 1.67). Here, S is defined as the ratio of a CS dose to the minimal CS dose required to cause COPD with the parameters given in Table A in S1 File.

As shown in Fig 2(B), M_1_ along with I_α_ and I_6_ [Fig 3(B)] rises to a peak at 12 days of CS exposure and then decays until day 60 because of the suppression of IL-10 secreted mainly by M2, showing an acute inflammatory response to the CS exposure. These results are qualitatively consistent with experiments in mice [47]) (see Figure B in S1 File for comparison with mice experiments). This period of time can be referred as step 1 in the progression of COPD as proposed by Agusti *et al*. [2–3]. After step 1, however, the inhibitory effect of IL-10 on M1 (as well as proinflammatory cytokines) is counteracted by the production of M1 up-regulated by TNF-α, TD, and INF-γ (Fig 1). It turns out that M_1_ is increased (but slowly) up to day 180 [Fig 2(B)]. This time period is referred as step 2 in the COPD progression [2, 3]. During this period, D_C_ along with I_12_ (data not shown), I_6_ and I_β_ [Fig 3(B)], and I_21_ (data not shown) increases slowly and gradually, resulting in the slow productions of T_1_, T_17_, and T_8_, respectively. Consequently, T_D_ rises gradually during this period. After step 2, innate proinflammatory network elements start to go up quickly as M1 predominates over M2 again [Fig 2(A)]. In particular, I_6_ is increased more rapidly than I_α_ [Fig 3(A)], enhancing the production of T_17_ and T_8_ and allowing the adaptive immunity to play an increasingly important role in response to the CS exposure and in tissue damage (Fig 4). Notably, T_8_ goes up more quickly than T_1_ and T_17_ [Fig 4(A)], dominating in the late phase in the COPD progression. Eventually, T_D_ together with the immune cells and cytokines goes to a steady state (stable COPD) as shown in Fig 4(B). The results for the above immune cells and cytokines at the steady state are listed in Table B in S1 File. Our results are in agreement with experiments [55–63].

**Fig 5:**
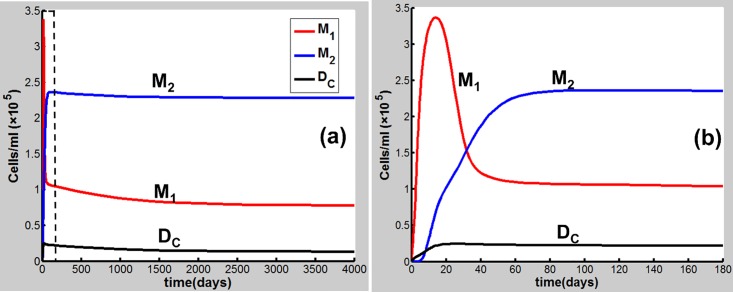
CS-induced (S = 0.7) population dynamics. Dynamics of M_1_, M_2_ and D_C_ over a time period of (a) 4000 days and (b) 180 days [the dashed square region in (a)].

**Fig 6:**
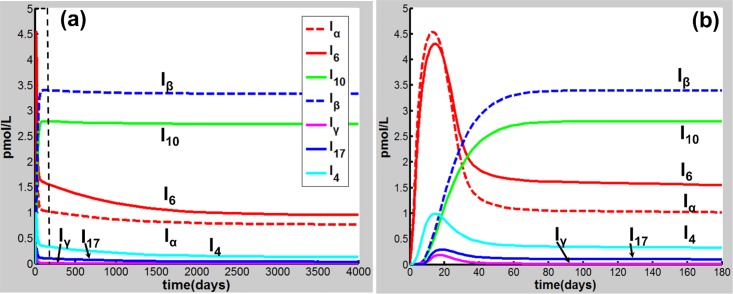
CS-induced (S = 0.7) population dynamics. Dynamics of I_α_, I_6_, I_10_, I_β_, I_γ_, I_17_ and I_4_ over a time period of (a) 4000 days and (b) 180 days [the dashed square region in (a)].

**Fig 7:**
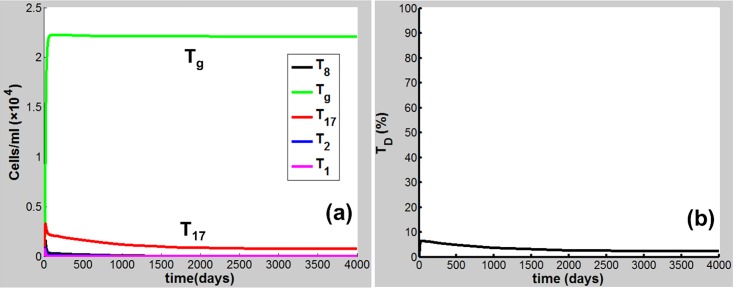
CS-induced (S = 0.7) population dynamics. Dynamics of (a) T_8_, T_g_, T_17_, T_2_, and T_1_, and (b) T_D_.

When S decreases to be less than unity, e.g., S = 0.7, the network exhibits a different dynamical feature. Upon CS exposure (S = 0.7), the network dynamical behaviors [Figs 5(B) and 6(B)] are similar (with smaller amplitudes) to those at S = 1.67 in the acute phase [Figs 2(B) and 3(B)]. After step 1, however, M_1_ together with other proinflammatory elements including I_α_ and I_6_ decreases to a low-level steady state (Figs 5 and 6) owing to the suppression of IL-10. Consequently, T_D_ maintains at a rather low level (<5%) when the system reaches a steady state (Fig 7), indicating that COPD does not occur. Compared to the case of S = 1.67 (Figs 2–4), M_2_ (along with T_g_, I_10_, and I_β_) (Figs 5–7) starts to predominate over M_1_ (with other proinflammatory components) during step 2 and remains predominant thereafter to a steady state where COPD does not occur.

The results for the cases of S = 1.67 and S = 0.7 indicate that during the transition from the innate to the adaptive immunity, when M_1_ predominates over M_2_, the system would proceed to high-grade chronic inflammation and eventually toward COPD (Figs 2–4); while M_2_ (Fig 5) [Treg (Fig 7A)] is predominant, the acute inflammation turns into the low-grade chronic inflammation (Fig 6), and COPD does not occur [Fig 7(B)].

## Cessation of Cigarette Smoking

**Fig 8:**
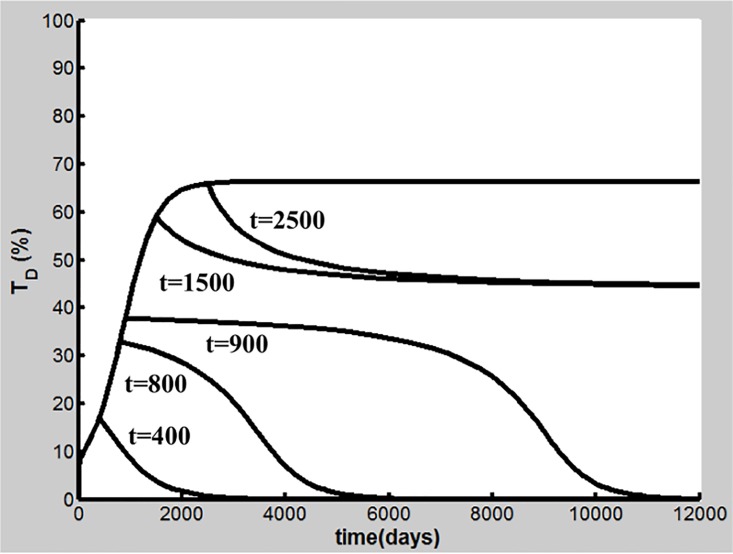
Effects of cigarette smoking cessation on T_D_ dynamics. Smoking cessation occurs after t = 400, 800, 900, 1500, and 2500 days of CS exposure, respectively.

Cigarette smoking cessation is considered a most important intervention to reduce COPD progression [10]. The dynamics of T_D_ is shown in Fig 8 as cigarette smoking cessation occurs after different days of CS exposure (S = 1.67) with the parameters in Table A in S1 File. Our simulations demonstrate that when early smoking cessation happens before 920 days of CS exposure, T_D_ falls to the baseline and COPD is prevented (Fig 8). Smoking cessation starting after day 920 leads to the reduction of T_D_ to some extent, but COPD and inflammatory responses persist (Figure C in S1 File). Our results are qualitatively consistent with experimental and clinical observations [64–66]. Analysis of positive feedback loops will elucidate further these effects of smoking cessation as discussed later.

### Susceptibility to COPD

As mentioned above, although CS is the major risk factor for COPD, only about 20–30% of chronic smokers are susceptible to the disease, suggesting that susceptibility of smokers to COPD varies [1, 7, 9]. This implies that parameters governing the dynamics of T_D_ as well as other important network elements in the model can change among smokers with different levels of COPD susceptibility. Importantly, effects of cigarette smoking cessation can be different among smokers with variable susceptibility [65]. While quitting smoking can prevent COPD in some patients (i.e., reversible susceptible smokers), smoking cessation fails to slow or stop COPD progression in others (severely susceptible smokers) [2, 66].

**Fig 9:**
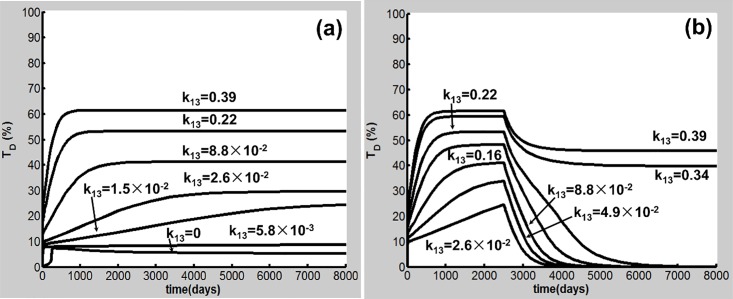
Dynamics of T_D_ with different values of k_13_ and effects of cigarette smoking cessation. (a) k_13_< 2.6×10^−2^ ml/(cell day) corresponds to resistant smokers (T_D_<30%), while k_13_≥2.6×10^−2^ ml/(cell day) is associated with susceptible smokers. (b) Effects of smoking cessation after 2500 days of CS exposure. 2.6×10^-2^ml/(cell day) ≤ k_13_ < 0.31ml/(cell day) corresponds to reversible susceptible smokers and COPD is reversible. k_13_≥0.31 ml/(cell day) is associated with severely susceptible smokers. In this case, COPD is not reversible.

Our global sensitivity analysis shows that the T_D_ outcome is sensitive to a subset of parameters including k_13_, k_14_, and k_15_ and d_TD_ in Eq 12. Fig 9 illustrates how variations in k_13_ affect the transitions from resistant, reversible to severely susceptible smokers. Here, the CS dose (S = 1.67) is the same as that in the case shown in Figs 2–4. While k_13_ is varied, d_TD_ = 3.4×10^−3^ (1/day) and the values of the other parameters in Table A in S1 File are used. The results show that when k_13_ is less than 2.6×10^−2^ ml/(cell day),T_D_ remains at a low-level (<30%), exhibiting a COPD resistant feature seen in Fig 9(A). While k_13_ lies in a value range of 2.6×10^−2^ and 0.31 ml/(cell day), COPD occurs but smoking cessation leads to T_D_ decreasing to the baseline shown in Fig 9(B). In this case, COPD is reversible [2]. When k_13_ is larger than 0.31 ml/(cell day), T_D_ at the steady state is reduced to some extent but still remains at a high level (>30%) after smoking cessation at day 2500. A COPD patient in this case is severely susceptible [11]. Interestingly, for a severely susceptible smoker whose k_13_ is relatively large, the M1-induced destruction of lung tissue predominates. In this case, M1 is sufficient for the progression of COPD, consistent with experiments in mice [24, 25].

A well-recognized example for severely susceptibility is the severe deficiency of α-1 antitrypsin, which is the major inhibitor of neutrophil elastase. The severe α-1 antitrypsin deficiency is present in only 1–2% of individuals with COPD [67]. As neutrophil elastase functions to degrade macrophage elastase inhibitor of metalloproteinase-1, the severe α-1 antitrypsin deficiency significantly increases the tissue destruction activity of macrophage elastase [68]**.** Therefore, the overall effect of the severe α-1 antitrypsin deficiency is to significantly enhance the M1-induced T_D_ generation [68], corresponding to the case where k_13_ adopts a relatively large value for a severely susceptible smoker (Fig 9).

Similar results were obtained by varying k_14_ and k_15_ shown in Figures D-E in S1 File, respectively. Intriguingly, the changes in some parameters such as $k_{I_{6},M_{1}}$ in Eq 10, which do not affect directly on the T_D_ outcome, also lead to similar results for T_D_ dynamics, shown in Figure F in S1 File. Here, our modeling results demonstrate that similar T_D_ outcomes can result from variations of different parameters. As will be discussed later, these parameters may be associated with different mechanisms of CS-induced immune responses in COPD progression, implying that similar COPD phenotypes can be caused by different mechanisms (endotypes). This finding could be important for developing therapeutic methods to treat COPD, as also discussed below.

### *In Silico* Knockout Simulations for Identification of Important Network Elements in COPD Progression

**Fig 10:**
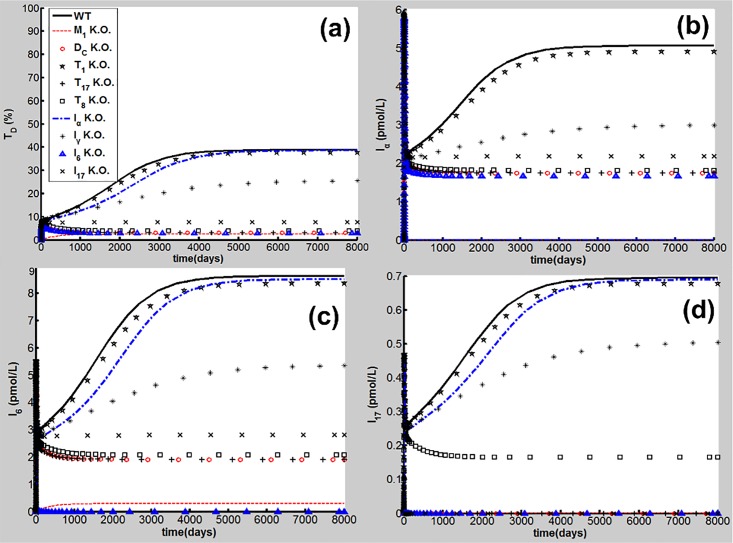
I*n silico* knockout simulations. (a) T_D_ dynamics, (b) I_α_ dynamics, (c) I_6_ dynamics, and (d) I_17_ dynamics. I*n silico* knockouts of M1 (red dashed line), DC (red circles), Th1 (black stars), Th17 (black plus), CD8^+^T (black squares), TNF-α (blue dash-and-dot line), INF-γ (black asterisk), IL-6 (blue triangles), and IL-17 (black cross) [wild type is denoted by WT (black solid line)].

To identify important network elements for CS-induced COPD, *in silico* knockout simulations for the proinflammatory elements, e.g., M1, DC, Th1, Th17, CD8^+^T cells and TNF-α, IL-6, IFN-γ, and IL-17 were conducted in the following discussion. Deletion of a network element in the model was performed by setting all parameters of the element and the rate to zero [69]. Here, the parameters used in the simulations are listed in Table A in S1 File [d_TD_ = 2.9×10^−3^ (1/day)] and the CS dose (S = 1.67) is the same as that shown in Figs 2–4. The results for the time courses of T_D_, and I_α_, I_6_ and I_17_ are presented in Fig 10.

As seen in Fig 10, M1 knockout results in significant reductions of T_D_ and proinflammatory signals to rather low levels despite continuous CS exposure. M1 not only secretes proinflammatory cytokines such as TNF-α, IL-6 and IL-12, but also causes tissue damage as discussed above (Fig 1). Our results are in line with experiments demonstrating that M1 is a determinant for CS-induced COPD [24].

DC knockout leads to the low-level outcomes of T_D_ and the adaptive immune signals such as I_17_ shown in Fig 10. The innate immune signals, e.g. I_α_ and I_6_, are reduced partially but still remain at a certain level. Upon deletion of Th1 there are no significant changes in the CS-induced TD and proinflammatory outcomes. This result is in agreement with clinical data for low Th1 population density [63], and mice experiments showing that CS induced significantly the production of Th17 rather than Th1 [70]. Deletion of Th17 or CD8^+^T cells leads to a significant reduction of T_D_ so that the progression of COPD is halted seen in Fig 10(A). However, the CS-induced proinflammatory signals such as I_α_ and I_6_ are reduced but still maintain at a relatively high level compared to those *in silico* experiment of M1 knockout. Different from the CD8^+^T deletion, the knockout of Th-17 leads to a reduction of I_17_ to the baseline, as shown in Fig 10(D). These results are in line with experiments in mice [70, 71].

TNF-α knockout simulations show that there is no significant reduction in T_D_ and other proinflammatory signals (Fig 10). This result is in line with clinical data and pharmaceutical studies discussed previously [6, 18, 19]. The IL-6 deletion allows the adaptive immune signals, T_17_, T_8_, I_21_ (data not shown) and I_17_ to maintain at the baseline level, and results in a significant reduction of T_D_ (<10%) at the steady state (Fig 10), whereas innate proinflammatory signals such as I_α_ maintains at a relatively high level (blue triangles in Fig 10). While the IL-17 knockout leads to the results similar to those in the deletions of Th17 and CD8^+^T, the IFN-γ deletion results in T_D_ (at the steady state) at a relatively high level (~32%, black asterisks in Fig 10).

### Loop Breaking Simulations to Identify Important Positive Feedback Loops in COPD Progression

**Fig 11:**
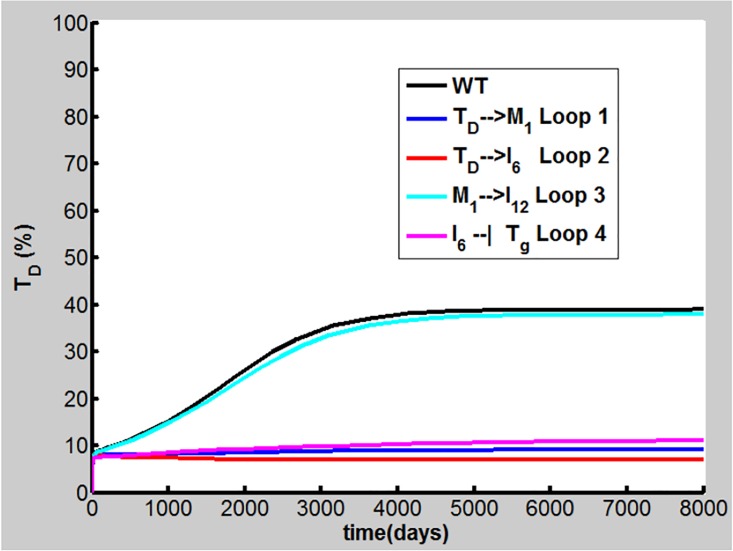
Loop breaking simulations. Loops 1, 2, 3, and 4 are broken on TD→M1 (blue line), TD→IL-6 (red line), M1→IL-12 (cyan line), and IL-6 ┫Treg (pink line), respectively (WT denotes wild type in black line) for dynamics of T_D_.

To investigate the importance of the positive feedback loops shown in Fig 1 in COPD progression, a feedback loop breaking approach is applied in the present study. Here, four aforementioned positive feedback loops, M1→TD→M1 (Loop 1), IL-6→Th17→ IL-17→TD→IL-6 (Loop 2), M1→IL-12→Th1→IFN-γ→M1 (Loop 3), and IL-6 ┫Treg→IL-10 ┫Th17→IL-17→TD→IL-6 (Loop 4), are explored by setting, e.g., k_3_ in Eq (A), k_I6, TD_ in Eq (J), and k_I12, M1_ in S1 File to zero, leading to the breaking of Loops 1–3 on TD→M1, TD→IL-6, and M1→IL-12, respectively. We set K_Tg, I6_ in Eq 6 to a very large value, e.g., 10^4^ to break Loop 4 on IL-6 ┫Treg in the calculation. Simulations were performed using our model with the parameters in Table A in S1 File (d_TD_ = 2.9×10^−3^/day). The results reveal that breaking Loop 1, 2, or 4 allows T_D_ to remain less than 10% (Fig 11), indicating that COPD does not occur. However, breaking Loop 3 has no profound effects on COPD progression (cyan line in Fig 11). This finding is consistent with virtual knockout of Th1 discussed above.

**Fig 12:**
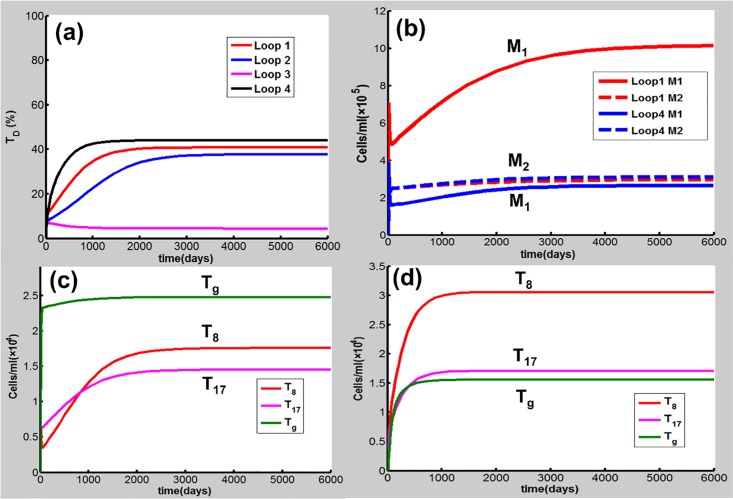
One of Loops 1, 2, 3, and 4 is activated while the others are broken. **(**a) Loop 1, 2, or 4 alone causes CODP while Loop 3 does not. (b) As Loop 1 is activated, M_1_ (red solid line) predominates over M_2_ (red dashed line). While Loop 4 is activated, both M_1_ (blue solid line) and M_2_ (blue dashed line) are relatively low. (c) In the case where Loop 1 is activated, T_g_ is predominant over T_8_ or T_17_. (d) The activation of Loop 4 leads to the predominance of T_8_ and T_17_ over T_g_.

Loops 1, 2 and 4, which all include the TD node, can amplify the immune response to CS and enhance the TD outcome when they are activated. As Loop 1, 2 or 4 is necessary for COPD progression under the condition given in Table A in S1 File discussed above, we also carried out simulations to investigate whether one of these loops is sufficient for COPD when other positive feedback loops are broken. Here, k_3_ = 1.9×10^6^ cell/(ml day), k_I6,TD_ = 22.0pmol/(cell day), and K_Tg,I6_ = 2.3 pmol/L are used for Loop 1, 2, and 4, respectively. The other parameter values are given in Table A in S1 File (d_TD_ = 2.9×10^−3^/day). The results show that activation of Loop 1, 2, or 4 can lead to COPD [Fig 12(A)]. Again, Loop 3 alone is not able to drive COPD progression by modification of k_I12, M1_.

It is of interest to note that Loops 1, 2 and 4 play important but different roles in the CS-induced immune response in COPD progression as shown in Fig 12. The activation of Loop 1 results in a marked predominance of M1 over M2, thus responsible for the innate immune response [Fig 12(B)]. In this case, T_g_ is predominant over T_8_ or T_17_ [Fig 12 (C)]. The activation of Loop 4, as well as Loop 2 (data not shown), drives Th17 and CD8^+^ to predominate over Treg, responsible for the adaptive immune response [Fig 12(D)]. In this case, both M_1_ and M_2_ are relatively low [Fig 12(B)]. These results demonstrate that there exist different cellular and molecular mechanisms (endotypes) in COPD progression. In particular, these different endotypes (mechanisms) can lead to similar T_D_ (CODP) outcomes, highlighting the heterogenous nature of COPD. This finding would be of particular importance in treatment of COPD using personalized medicine and target therapy [72] as discussed below.

## Discussion

The molecular and cellular mechanisms of the CS-induced immune response in COPD progression are not completely understood. In particular, the issues regarding the dynamics of CS-induced immune response in COPD, the effects of cigarette smoking cessation, and the disease susceptibility remain to be elucidated [1–2]. As COPD is a chronic and progressive inflammatory disease whose dynamical time scale is usually very long (over 20 years of CS exposure [11]), it would be extremely difficult for real-time measurements in the clinic or the laboratory. The main objective of this paper is to use computer modeling and simulation to address these issues. As discussed above, the nodes in our network model, which bears a multiscale nature, represent the cytokines, immune cells and damaged tissues (TD) whose dynamics characterizes the progression of COPD. The interactions between these nodes are often highly nonlinear and can be described using the Hill functions. The population dynamics of the network elements are described by a set of DOEs with parameters, whose values are known or estimated from established literature. For those no experimental data are available, we performed the global sensitivity analysis to obtain order-of-magnitude estimates.

The results in this work demonstrate that CS-induced COPD development is a multi-step process involving both innate and adaptive immune responses. In the early acute phase of CS exposure, innate immune response predominates. During the transition from the innate to the adaptive immunity, if M_1_ predominates over M_2_, the system proceeds to high-grade chronic inflammation and eventually toward COPD where the adaptive immunity play a dominant role (Figs 2–4). However, when M_2_ (Treg) is predominant over M1 (Th17 and CD8^+^ T), the acute inflammation turns into the low-grade chronic inflammation, and COPD does not occur (Fig 5–7).

CS inhalation has been considered the major risk factor for COPD, but only about 20–30% chronic smokers develop the disease, suggesting that cigarette smokers have different levels of COPD susceptibility [1, 9]. Our simulations disclose that there exist three types of smokers according to their COPD susceptibilities, i.e., resistant, reversely susceptible and severely susceptible smokers. While long-term CS inhalation can cause just low levels of chronic inflammation in resistant smokers but without COPD, susceptible smokers can develop COPD eventually under the same CS exposure conditions as shown in Fig 9. After cigarette smoking cessation, COPD can be prevented in reversible susceptible smokers, but the disease cannot be fully reversed in severely susceptible smokers (Fig 9). The sensitivity analysis in this work has identified a subset of parameters in the model that govern the dynamical behaviors of COPD and describe the different disease susceptibilities of different smokers as shown in Fig 9 and Figures D-F in S1 File.

The network model in this paper involves multiple immune cells, cytokines and lung tissues, forming multiple proinflammatory and anti-inflammatory/regulatory pathways with several positive feedback loops. The *in silico* knockout experiments performed in this study have identified several important proinflammatory elements including IL-6, IL-17 cytokines, and M1, DC, Th17, CD8^+^T cells. It is intriguing to note that despite high concentrations of TNF-α often found in COPD smokers, our present study shows that this cytokine may not play a significant role in the progression of COPD. This finding is consistent with biopharmaceutic and clinical studies [6, 18, 19]. The feedback loop breaking simulations demonstrate that Loops 1, 2 and 4, which all involve the TD node, play important but different roles in the COPD progression. Activation of Loop 1, which enhances the M1 and TD productions, can promote the M1/M2-type COPD while Loops 2 and 4 contribute to the (Th17+CD8^+^T)/Treg-type disease where IL-6 and IL-17 are key molecules for the disease progression. These results indicate that COPD can be heterogeneous and can result from different molecular and cellular mechanisms.

The reason why inflammation persists in COPD patients even after long-term smoking cessation is currently unknown [2]. The above loop breaking results may provide novel insight into the aforementioned smoking cessation effects. After cigarette smoking cessation, the disease remains if these positive feedback loops continuously work to cause the tissue damage. But when TD is reduced due to smoking cessation to such an extent that these positive feedback loops are unable to drive tissue damage further, COPD will be suppressed.

The feedback loop breaking simulations are consistent with the *in silico* knockout experiments in this study, together identifying the network inflammatory determinants for CODP progression. For example, IL-6 is not only a product from Loop 1, but also a key component of both Loops 2 and 4. IL-6 is an important proinflammatory factor for synthesis of acute phase proteins such as C-reactive protein, which is associated with several acute and chronic inflammatory diseases including COPD [15, 73, 74]. This cytokine is also identified as a major regulator of the balance between Treg and Th17 (CD8^+^T) cells as seen in our above discussions. As IL-6 plays an important role in COPD progression, it has recently been recognized as a potential target for COPD [73, 74]. Another example is that IL-17 as well as Th-17 is identified as a key component for COPD. Targeting IL-17 and Th-17 has become a promising strategy for treatment of the disease [75]. However, COPD is a complex and heterogeneous disorder, e.g. similar clinical phenotypes can come from different endotypes, which are associated with different molecular and cellular mechanisms, as shown in our above modeling analysis. It is critical to identify the molecular and cellular disease mechanisms, by which subtypes of COPD patients are defined. As such, future treatment options would target the identified endotypes using available anti-inflammatory drugs [72]. For this purpose, specific biomarkers of these endotypes would be particularly useful. Our modeling study offers a possible approach to probe endotype biomarkers for COPD and provides novel insight into this personalized medicine strategy as discussed above.

As we focus on the CS-induced immune response in the progression towards stable COPD in this work, other pathogenic mechanisms including exaggerated proteolytic activity, resulting from an imbalance between protease and antiprotease, and excessive oxidative stress from an oxidant-antioxidant imbalance [7] are implicitly involved, for example, in the representation of the M1→TD process as discussed above. Disruption of the balance between cell death and repair is also included indirectly in the TD component. These highly coarse-grained representations can be extended in more detail. Indeed, such studies have been undertaken recently in references [76–77]. However, the adaptive immunity has not been investigated and the insight into the cellular and molecular mechanisms of COPD is incomplete in these studies [76,77].

Although cigarette smoking is the main risk factor for COPD, other factors can also play an important role in disease development and progression [78]. For example, as almost every component of the immune system undergoes age-associated changes, aging is a risk factor for developing COPD [78]. An exacerbation of COPD (ECOPD) is defined as an acute event characterized by a worsening of the patient’s respiratory symptoms that is beyond normal day-to-day variations and leads to a change in medication, responsible for substantial COPD mortality [79]. Viral or bacterial infections are the main causes of ECOPD, leading to an acute flare-up of inflammation in the lung with stable COPD. Interestingly, mechanical forces of lung tissues have been shown to contribute to COPD progression by allowing rupture of tissue elements which directly leads to increased airspaces and this progression can go on even after smoking cessation [80]. It is important to point out that our current network model focusing on CS-induced COPD does not take these factors into account. Elucidating the roles of these factors in COPD progression is beyond the scope of this article. Possible extensions of the present work to incorporate these factors into the network model will be investigated in future studies.

It is of great interest to note that COPD and lung cancer are frequently induced by cigarette smoking, but these two disorders show opposite phenotypes [81, 82]. While COPD is featured by excessive lung injury and airway epithelial cell death, lung cancer is caused by unregulated proliferation of epithelial cells [82]. Numerous epidemiological studies have linked the presence of COPD with increased lung cancer incidence, however, the molecular and cellular links between these two diseases remain obscure [5, 82–84]. Our network modeling research along this line remains for future.

## Conclusion

In this work, we develop a network model for the dynamics of CS-induced immune response in COPD progression. Using this model, computer simulations are performed for the investigations of smoking cessation effects and susceptibility of smokers to COPD, and the identification of important network elements in COPD progression. Our modeling study identifies several positive feedback loops that play important but different roles in COPD progression. The computational results in this study are consistent with laboratory and clinical observations, providing novel insight into the cellular and molecular mechanisms of CS-induced COPD.

## Supporting Information

S1 File Equations A-G, Tables A-B, and Figures A-F are included in supporting information.
(DOCX)
