# Supplementary material for: Probing Cellular and Molecular Mechanisms of Cigarette Smoke-Induced Immune Response in the Progression of Chronic Obstructive Pulmonary Disease Using Multiscale Network Modeling
Source: PLoS One. 2016 Sep 26;11(9):e0163192. doi: 10.1371/journal.pone.0163192 (PMC5036797; doi:10.1371/journal.pone.0163192)
Supplement: S1 File — Equations A-G, Tables A-B, and Figures A-F are included in supporting information. (DOCX) [file pone.0163192.s001.docx]

**Supporting Information**

**Probing Cellular and Molecular Mechanisms of Cigarette Smoke-Induced Immune Response in the Progression of Chronic Obstructive Pulmonary Disease Using Multiscale Network Modeling**

Zhichao Pan, Haishan Yu, Jie-Lou Liao*

Department of Chemical Physics, University of Science and Technology of China, 96 Jinzhai Road, Hefei, Anhui Province, People’s Republic of China, 230026

*E-mail: [liaojl@ustc.eud.cn](mailto:liaojl@ustc.eud.cn)

Equations A-G for the population dynamics of cytokines, TNF-α, IL-12, IFN-γ, IL-17, IL-21, IL-4, and TGF-β are given, respectively, by

$\frac{dI_{\alpha}}{dt}=k_{I_{\alpha},M_{1}}M_{1}\frac{1}{1+\left(I_{10}/K_{M_{1},I_{10}}\right)^{2}}-d_{I_{\alpha}}I_{\alpha}$ (A)

$\frac{dI_{12}}{dt}=(k_{I_{12},M_{1}}M{}_{1}+k_{I_{12},D_{c}}D_{c})\frac{1}{1+\left(I_{10}/K_{I_{12},I_{10}}\right)^{2}}-d_{I_{12}}I_{12}$ (B)

$\frac{dI_{\gamma}}{dt}=(k_{I_{\gamma},T_{1}}T{}_{1}+k_{I_{\gamma},T_{8}}T_{8})\frac{1}{1+\left(I_{10}/K_{I_{\gamma},I_{10}}\right)^{2}}-d_{I_{\gamma}}I_{\gamma}$ (C)

$\frac{dI_{17}}{dt}=k_{I_{17},T_{17}}T_{17}\frac{1}{1+\left(I_{10}/K_{I_{17},I_{10}}\right)^{2}}-d_{I_{17}}I_{17}$ (D)

$\frac{dI_{21}}{dt}=k_{I_{21},T_{17}}T_{17}\frac{1}{1+\left(I_{10}/K_{I_{21},I_{10}}\right)^{2}}-d_{I21}I_{21}$ (E)

$\frac{dI_{4}}{dt}=k_{I_{4},T_{2}}T_{2}\frac{1}{1+\left(I_{10}/K_{T_{2},I_{10}}\right)^{2}}+k_{I_{4},T_{D}}T_{D}\frac{1}{1+\left(I_{10}/K_{T_{D},I_{10}}\right)^{2}}-d_{I_{4}}I_{4}$ (F)

$\frac{dI_{\beta}}{dt}=k_{I_{\beta},M_{2}}M{}_{2}+k_{I_{\beta},T_{g}}T_{g}-d_{I_{\beta}}I_{\beta}$ (G)

**Table A. Parameters for the equations describing network dynamics of CS-induced immune response**

| Name | Value | Unit | Reference |
| --- | --- | --- | --- |
| k1 | 0.54 | 10^5^cell/ml/day | Estimated in this work |
| k2 | 1.50 | 10^5^cell/ml/day | Estimated from [1],[2],[3] |
| k3 | 6.35 | 10^5^cells/ml/day | Estimated |
| k4 | 1.20 | 10^5^cell/ml/day | [1],[2],[3] |
| k5 | 0.067 | 10^5^cell/ml/day | Estimated |
| k6 | 1.73 | 10^5^cell/ml/day | [2],[3],[4] |
| k7 | 0.60 | 10^4^ /day | [3],[4],[5] |
| k8 | 0.41 | 10^4^ /day | [3],[4],[5] |
| k9 | 3.05 | 10^4^ /day | [5] |
| k10 | 0.45 | 10^4^ /day | [5],[6] |
| k11 | 0.91 | 10^5^cell/(ml day) | [5],[7] |
| k12 | 4.3×10^-5^ | 1/day | Estimated |
| k13 | 2.4×10^-3^ | ml/(cell day) | Estimated |
| k14 | 1.9×10^-2^ | ml/(cell day) | Estimated |
| k15 | 3.9×10^-3^ | 1/day | Estimated |
| $k_{1}^{p}$ | 0.31 | 10^4^/day | [2],[8] |
| $k_{2}^{p}$ | 0.19 | 10^4^/day | [2],[8] |
| $k_{17}^{p}$ | 0.15 | 10^4^/day | [2],[8] |
| $k_{8}^{p}$ | 0.15 | 10^4^/day | [2],[8] |
| $k_{g}^{p}$ | 0.03 | 10^4^/day | [2],[8] |
| $k_{I_{\alpha},M_{1}}$ | 2.25 | pmol/(cell day) | [2] |
| $k_{I_{12},M_{1}}$ | 4.5×10^-3^ | pmol/(cell day) | [2],[3] |
| $k_{I_{12},D_{C}}$ | 1.1×10^-2^ | pmol/(cell day) | [2],[3] |
| $k_{I_{\gamma},T_{1}}$ | 0.72 | Pmol/(cell day) | [2],[3] |
| $k_{I_{\gamma},T_{8}}$ | 0.72 | pmol/(cell day) | [2],[3] |
| $k_{I_{6},M_{1}}$ | 0.20 | pmol/(cell day) | Estimated |
| $k_{I_{6},T_{D}}$ | 2.21 | pmol/(cell day) | Estimated |
| $k_{I_{17},T_{17}}$ | 0.64 | pmol/(cell day) | [9],[10] |
| $k_{I_{21},T_{17}}$ | 14.4 | pmol/(cell day) | [11] |
| $k_{I_{4},T_{D}}$ | 1.56 | pmol/(cell day) | Estimated |
| $k_{I_{4},T_{2}}$ | 0.83 | pmol/(cell day) | [8] |
| $k_{I_{10},M_{2}}$ | 0.75 | pmol/(cell day) | [2],[9] |
| $k_{I_{10},T_{g}}$ | 0.90 | pmol/(cell day) | [2],[9] |
| $k_{I_{\beta},M_{2}}$ | 1.44×10^-2^ | pmol/(cell day) | [7] |
| $k_{I_{\beta},T_{g}}$ | 0.17 | pmol/(cell day) | [7] |
| K1 | 10.1 | pmol/L | Estimated |
| K2 | 0.1 | pmol/L | [2],[8] |
| K3 | 1.3 | pmol/L | Estimated |
| K4 | 0.5 | pmol/L | [8] |
| K5 | 3.8 | pmol/L | Estimated |
| K6 | 2.1 | pmol/L | [4] |
| K7 | 3.0 | pmol/L | Estimated |
| K8 | 50.1 | pmol/L | Estimated |
| K9 | 0.3 | pmol/L | [2] |
| K10 | 10.0 | pmol/L | Estimated |
| K11 | 0.2 | pmol/L | Estimated |
| K12 | 2.2 | pmol/L | Estimated |
| K13 | 0.8 | pmol/L | Estimated |
| K14 | 0.4 | pmol/L | Estimated |
| K15 | 0.7 | pmol/L | Estimated |
| K16 | 1.2 | pmol/L | Estimated |
| $K_{T_{1}}$ | 1.0 | pmol/L | [4] |
| $K_{T_{2}}$ | 0.3 | pmol/L | [4] |
| $K_{T_{17}}$ | 1.3 | (pmol/L)^2 | Estimated |
| $K_{T_{8}}$ | 5.1 | pmol/L | Estimated |
| $K_{T_{g}}$ | 2.0 | pmol/L | [7] |
| $K_{T_{1},I_{10}}$ | 1.5 | pmol/L | Estimated |
| $K_{T_{2},I_{10}}$ | 1.0 | pmol/L | Estimated |
| $K_{T_{17},I_{10}}$ | 0.9 | pmol/L | Estimated |
| $K_{T_{8},I_{10}}$ | 4.0 | pmol/L | Estimated |
| $K_{T_{g},I_{6}}$ | 10.4 | pmol/L | Estimated |
| $K_{T_{1},I_{10}}^{p}$ | 2.1 | pmol/L | Estimated |
| $K_{T_{2},I_{10}}^{p}$ | 1.5 | pmol/L | Estimated |
| $K_{T_{17},I_{10}}^{p}$ | 1.0 | pmol/L | Estimated |
| $K_{T_{8},I_{10}}^{p}$ | 2.1 | pmol/L | Estimated |
| ${K^{P}}_{T_{g},I_{6}}$ | 0.1 | pmol/L | Estimated |
| $K_{I_{\alpha},I_{10}}$ | 4.5 | pmol/L | Estimated |
| $K_{I_{12},I_{10}}$ | 5.1 | pmol/L | [2],[8] |
| $K_{I_{\gamma},I_{10}}$ | 3.0 | pmol/L | [2] |
| $K_{M_{1},I_{10}}$ | 6.2 | pmol/L | Estimated |
| $K_{T_{D},I_{10}}$ | 7.0 | pmol/L | Estimated |
| $K_{I_{17},I_{10}}$ | 2.8 | pmol/L | Estimated |
| $K_{I_{21},I_{10}}$ | 4.0 | pmol/L | Estimated |
| $K_{I_{4},I_{10}}$ | 1.8 | pmol/L | Estimated |
| $K_{I_{10},I_{10}}$ | 80.4 | pmol/L | [2] |
| $d_{M_{1}}$ | 0.60 | 1/day | [1],[2],[3] |
| $d_{M_{2}}$ | 0.24 | 1/day | [1],[2],[3] |
| $d_{D_{C}}$ | 0.61 | 1/day | [2],[12] |
| $d_{T_{1}}$ | 9.92 | 1/day | [1],[5] |
| $d_{T_{2}}$ | 9.91 | 1/day | [1],[5] |
| $d_{T_{17}}$ | 3.04 | 1/day | [1],[5] |
| $d_{T_{8}}$ | 9.13 | 1/day | [1],[5] |
| $d_{T_{g}}$ | 3.05 | 1/day | [1],[5] |
| $d_{I_{\alpha}}$ | 1.65×10^4^ | 1/day | [13],[14] |
| $d_{I_{12}}$ | 33.6 | 1/day | [15],[16] |
| $d_{I_{\gamma}}$ | 78.1 | 1/day | [15],[17] |
| $d_{I_{6}}$ | 186 | 1/day | [18],[19] |
| $d_{I_{17}}$ | 62.4 | 1/day | [9],[17] |
| $d_{I_{21}}$ | 165 | 1/day | [10],[20] |
| $d_{I_{4}}$ | 83.1 | 1/day | [21],[22] |
| $d_{I_{10}}$ | 135 | 1/day | [9],[23] |
| $d_{I_{\beta}}$ | 20.8 | 1/day | [24] |
| $d_{T_{D}}$ | 1.7×10^-3^ | 1/day | Estimated |

**Table B. Cell density and cytokine concentrations at the steady state from the simulations compared to experiments**

| Name | Experimental  data | Modeling  results | Unit | Reference |
| --- | --- | --- | --- | --- |
| M_1_ | 8.82 | 7.51 | 10^5^ cells/ml | [1],[ 25] |
| M_2_ | 3.78 | 3.80 | 10^5^ cells/ml | [1], [25] |
| D_C_ | 1.50 | 1.64 | 10^5^ cells/ml | [12] |
| T_8_ | 0.49 | 0.43 | 10^5^ cells/ml | [1],[ 5], [6] |
| T_17_ | 0.20 | 0.24 | 10^5^ cells/ml | [1],[ 5], [6] |
| T_g_ | 0.19 | 0.19 | 10^5^ cells/ml | [1],[ 5], [6] |
| T2 | 0.067 | 0.060 | 10^5^ cells/ml | [1],[ 5], [6] |
| T1 | 0.053 | 0.043 | 10^5^ cells/ml | [1],[ 5], [6] |
| I_α_ | 5.34 | 6.50 | pmol/L | [13] |
| I_12_ | 0.82 | 0.91 | pmol/L | [26] |
| I_γ_ | 1.83 | 1.91 | pmol/L | [26] |
| I_6_ | 15.14 | 12.66 | pmol/L | [13] |
| I_17_ | 0.82 | 1.03 | pmol/L | [27] |
| I_21_ | 12.90 | 12.45 | pmol/L | [11] |
| I_4_ | 2.81 | 2.88 | pmol/L | [28] |
| I_10_ | 2.74 | 3.38 | pmol/L | [9] |
| I_β_ | 4.20 | 4.14 | pmol/L | [26] |

**References**

1. Domagala-Kulawik J, Maskey-Warzechouska M, Kraszewsk I, Chazan R (2003) The cellular composition and macrophage phenotype in induced sputum in smokers and ex-smokers with COPD. Chest 123:1054-1059.

2. Day J, Friedman A, Schlesinger LS (2009) Modeling the immune rheostat of acrophages in the lung in response to infection. PNAS 106:11246-11251.

3. Wiggingon, JE, Kirschner D (2001) A model to predict cell-mediated immune regulatory mechanisms during human infection with mycobacterium tuberculosis. J Immunol 166:1951-1967.

4. Marino S, Kirschner DE (2004) The human immune response to mycobacterium tuberculosis in lung and lymph node. J Theor Biol 227:463-486.

5. Vargas-Rojas MI, Ramirez-Venegas A, Limon-Camacho L, Ochoa L, Hernandez- Zenteno R, Sansores RH (2011) Increase of Th17 cells in peripheral blood of patients with chronic obstructive pulmonary disease. Respir. Med 105:1648-1654.

6. Domagala-Kulawik J, Hoser G, Dabrowska M, Chazan R (2007) Increased proportion of Fas positive CD8+ cells in peripheral blood of patients with COPD. Respir. Med 101:1338-1343.

7. Liao KL, Bai XF, Friedman A (2014) Mathematical modeling of interleukin-35 promoting tumor growth and angiogenesis. PLoS One 9:e110126.

8. Friedman A, Turner J, Szomolay B (2008) A model on the influence of age on immunity to infection with mycobacterium tuberculosis. E*xper Geront* 43:275-285.

9. Zhang L, Cheng ZS, Liu WM, Wu KS (2013) Expression of interleukin (IL)-10, IL-17(A) and IL-22 in serum and sputum of stable chronic obstructive pulmonary disease patients. COPD: J COPD 10:459-465.

10. Thompson JA, Curti BD, Redman BG, Bhatia S, Weber JS, Agarwala SS, et al. (2008) Phase I study of recombinant interleukin-21 in patients with metastatic melanoma and renal cell carcinoma. J. Clin Oncol 26: 2034-2039.

11. Yerkovich ST, Hales BJ, Carroll ML, Burel GJ, Towers MA, Smith DJ, et al. (2012) Reduced rhinovirus-specific antibodies are associated with acute exacerbations of chronic obstructive pulmonary disease requiring hospitalization. BMC Pulmon Med 12:37.

12. D’hulst AI, Vermaelen KY, Brusselle GG, Joos GF, Pauwels RA (2005) Time course of cigarette smoke-induced pulmonary inflammation in mice. Eur Respir J 26:204-213.

13. Hacievliyagil SS, Gunen H, Mutlu LC, Karabulut AB, Temel I (2006) Association between cytokines in induced sputum and severity of chronic obstructive pulmonary disease. Respir Med 100:846-854.

14. MacKenzie S, Fernandez-Troy N, Espel E (2002) Post-transcriptional regulation of TNF-a during in vitro differentiation of human monocytes/macrophages in primary culture. J Leukocyte Biol 71:1026-1032.

15. Portielje JE, Kruit WH, Eerenberg AJ, Schuler M, Sparreboom A, Lamers CH et al. (2001) Interleukin 12 induces activation of fibrinolysis and coagulation in humans. Br J Haematol 112: 499-505.

16. Hamza TJ, Barnett JB, Li BY (2010) Interleukin 12 a key immunoregulatory cytokine in infection applications. Int J Mol Sci 11:789-806.

17. Cowan J, Pandey S, Filion LG, Angel JB, Kumar A, Gameron DW (2011) Comparison of interferon-γ-， interleukin (IL)-17- and IL-22-expressing CD4 T cells, IL-22-expressing granulocytes and proinflammatory cytokines during latent and active tuberculosis infection. Clin Exp Immunol 167:317-329.

18. Peters M, Jacobs S, Ehlers M, Vollmer P, Mullberg J, Wolf E et al. (1996) The function of the soluble interleukin 6 (IL-6) receptor in vivo: sensitization of human soluble IL-6 receptor transgenic mice towards IL-6 and prolongation of the plasma half-life of IL-6. J Exp Med 183:1399-1406.

19. Ridker PM, Rifai N, Stampfer MJ, Hennekens CH (2000) Plasma concentration of interleukin-6 and the risk of future myocardial infarction among apparently healthy men. Circulation 101:1767-1772.

20. Bhatia S, Curti B, Ernstoff MS, Gordon M, Heath EI, Miller WH, et al. (2014) Recombinant interleukin-21 plus sorafenib for metastatic renal cell carcinoma: a phase 1/2 study. J Immunol Therapy of Cancer 2:2.

21. Shen BJ, Hage T, Sebald W (1996) Global and local determinants for the kinetics of interleukin-4/interleukin-4 receptor α chain interaction: a biosensor study employing recombinant interleukin-4-binding protein. Eur J Biochem 240:252-261.

22. Steinke JW, Borish L (2001) Th2 cytokines and asthma, Interleukin-4: its role in the pathogenesis of asthma, and targeting it for asthma treatment with interleukin-4 receptor antagonists. Respir Res 2:66-70.

23. Edwards JP, Zhang X, Frauwirth KA, Mosser DM (2006) Biochemical and functional characterization of three activated macrophage populations. J Leukocyte Biol 80:1298-1307.

24. Kaminska B, Wesolowska A, Danilkiewicz M (2005) TGF beta signaling and its role in turmor pathogenesis. Acta Biochim Pol 52:329-337.

25. Kunz LI, Lapperre TS, Snoeck-Stroband JB, Budulac SE, Timens W, Wijngaarden SV, et al. (2011) Smoking status and anti-inflammatory macrophages in bronchoalveolar lavage and induced sputum in COPD. Respir Res 12:34.

26. Kalathil SG, Lugade AA, Pradhan V, Miller A, Parameswaran GI, Sanjay S, et al. (2014) T-regulatory cells and programmed death 1+ T cells contribute to effector T-cell dysfunction in patients with chronic obstructive pulmonary disease. Am J Respir Crit Care Med 190:40-50.

27. Li XN, Pan X, Qiu D (2014) Imbalances of Th17 and Treg cells and their respective cytokines in COPD patients by disease stage. Int J Clin Exp Med 7:5324-5329.

28. Kim V, Cornwell WD, Oros M, Durra H, Criner GJ, Rogers TJ (2015) Plasma chemokine signature correlates with lung goblet cell hyperplasia in smokers with and without chronic obstructive pulmonary disease. BMC Pulmon Med 15:111.

**Figure A. Sensitivity analysis.** PRCC values for the parameters in Table A with an

absolute value of PRCC > 0.1.

**Figure B. Dynamics of macrophages (M_1_+M_2_) from the modeling simulation (black**

**solid line) compared with mice experiments (red solid circles).** Here, k_2_, k_3_ and K_4_

are 3.0 (10^5^ cell/ml/day), 20.6 (10^5^ cell/ml/day) and 0.28 (pmol/L), the values of the

other parameters are the same as in Table S1 and S=1.67. The mice experimental data

are taken form ref. 47 in the main text.

**Figure C. Effects of cigarette smoking cessation.** Population dynamics of I_6_, I_α_, and I_17_

as smoking cessation occurs after 2500 days of CS exposure.

**Figure D. T_D_ dynamics with different k_14_ values and effects of cigarette smoking cessation.** (a) k_14_ <0.088 ml/(cell day) corresponds to resistant smokers, while k_14_ ≥ 0.088 ml/(cell day) is associated with susceptible smokers. (b) Effects of smoking cessation after 2500 days of CS exposure. COPD is reversible when 0.088ml/(cell day)≤ k_14_ < 0.18ml/(cell day). When k_14_ ≥ 0.18ml/(cell day), COPD is not reversible.

**Figure E. T_D_ dynamics with different k_15_ values and effects of cigarette smoking cessation.** (a) k_15_ < 0.020 ml/(cell day) corresponds to resistant smokers, while k_15_≥ 0.020 ml/(cell day) is associated with susceptible smokers. (b) Effects of smoking cessation after 2500 days of CS exposure. COPD is reversible when 0.020ml/(cell day) ≤ k_15_<0.083ml/(cell day). When k_15_≥0.083ml/(cell day), COPD is not reversible.


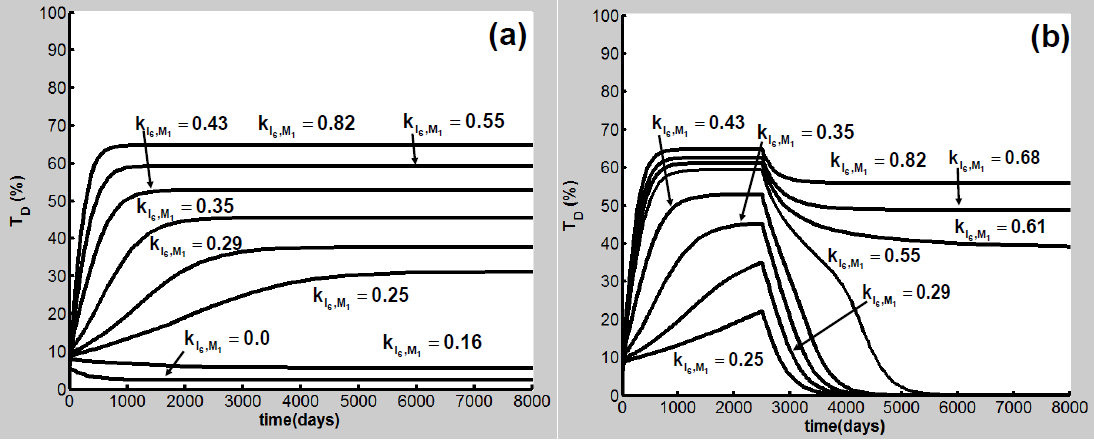


**Figure F. T_D_ dynamics with different values and effects of cigarette smoking**

**cessation.** (a) < 0.25 ml/(cell day) corresponds to resistant smokers, while 0.25 ml/(cell day) is associated with susceptible smokers. (b) Effects of smoking cessation after 2500 days of CS exposure. COPD is reversible when 0.25ml/(cell day) < 0.61ml/(cell day). When 0.61ml/(cell day), COPD is not reversible.
